# An Euler Graph-Based Path Planning Method for Additive Manufacturing Thin-Walled Cellular Structures of Continuous Fiber-Reinforced Thermoplastic Composites

**DOI:** 10.3390/polym17233236

**Published:** 2025-12-04

**Authors:** Guocheng Liu, Fei Wang, Qiyong Tu, Ning Hu, Zhen Ouyang, Wenting Wei, Lei Yang, Chunze Yan

**Affiliations:** 1 Hubei Key Laboratory of Advanced Technology for Automotive Components, Wuhan University of Technology, Wuhan 430074, China; 2 School of Automotive Engineering, Wuhan University of Technology, Wuhan 430074, China; 3 State Key Laboratory of Materials Processing and Die & Mould Technology, Huazhong University of Science and Technology, Wuhan 430074, China

**Keywords:** 3D printing, carbon fibers, Euler graph, honeycomb, path planning

## Abstract

Thin-walled cellular structures of continuous fiber-reinforced thermoplastic composites (CFRTPCs) have received much attention from both academics and industry due to their superior properties. Additive manufacturing provides an efficient solution for fabricating these thin-walled cellular structures of CFRTPCs. However, the process often requires cutting fiber filaments at jumping points during printing. Furthermore, the filament may twist, fold, and break due to sharp turns in the printing path. These issues adversely affect the mechanical properties of the additive manufactured part. In this paper, a Euler graph-based path planning method for additive manufacturing of CFRTPCs is proposed to avoid jumping and sharp turns. Euler graphs are constructed from non-Eulerian graphs using the method of doubled edges. An optimized Hierholzer’s algorithm with pseudo-intersections is proposed to generate printing paths that satisfy the continuity, non-crossing, and avoid most of the sharp turns. The average turning angle was reduced by up to 20.88% and the number of turning angles less than or equal to 120° increased by up to 26.67% using optimized Hierholzer’s algorithm. In addition, the generated paths were verified by house-made robot-assisted additive manufacturing equipment.

## 1. Introduction

**Figure 1:**
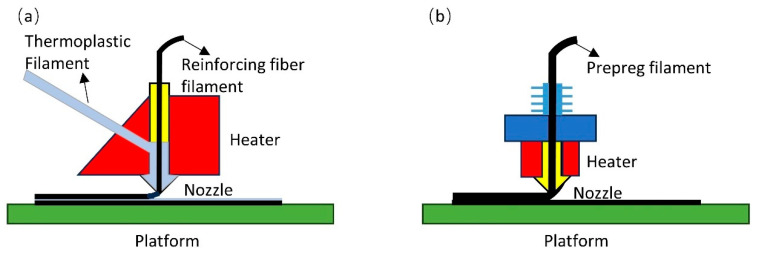
Schematic diagrams of the CFRTPCs AM methods: (**a**) in situ impregnation; (**b**) prepreg method.

Continuous fiber-reinforced thermoplastic composites (CFRTPCs) have attracted significant research focus in both academia and industry due to their excellent mechanical properties [1,2,3,4,5]. CFRTPCs components are developing towards the trend of lightweight, complexity, and integration. Additive manufacturing (AM) technology provides an efficient solution for designing large and complex components of CFRTPCs. In recent years, in situ impregnation [6,7,8,9] and prepreg methods [10,11,12] have been widely used in the AM processing of CFRTPCs. As illustrated in Figure 1, the in situ impregnation method separates the thermoplastic filament from the reinforcing fiber filament. The thermoplastic filament is heated in the print head, while the reinforcing fiber filament is impregnated with thermoplastic filament in the print head before being extruded and solidified through the nozzle. The prepreg filament method is to impregnate thermoplastic filament and fiber filament into a solidified filament outside the printing equipment in advance. The prepreg filament is used as a raw material for 3D printing. The algorithm mentioned in this paper can be applied to the above methods. Tian et al. [13] proposed an AM method for CFRTPCs based on the in situ impregnation. Continuous fibers were infiltrated and coated by a thermoplastic material inside the nozzle. The impregnated composite was extruded through the nozzle. This method enables printing for CFRTPCs. However, this in situ impregnation method had a short impregnation time and poor bonding between matrix and reinforcement materials [14]. Forming through the prepreg filament method can achieve better bonding between the matrix and reinforcement materials [15]. However, the mechanical properties of CFRTPCs parts were limited due to the poor interlayer bonding and high porosity of the printed parts [16]. Masahito et al. [3,17] proposed a 3D compaction printing technique for CFRTPCs. Compared with conventional fused deposition modeling, the flexural strength increased about 62% and the porosity was reduced from 10% to 3%. Applying pressure to the filament immediately after the printing can reduce voids and improve adhesion between the filaments. M. Bhatt et al. [18] proposed the advantages of using robots in AM. Robots could realize the multi-directional fabrication, conformal deposition, and large-scale AM. The house-made robot-assisted additive manufacturing equipment integrated the multiple degrees of freedom manufacturing and compaction processing so that the low porosity and high mechanical part can be printed.

Lightweight thin-walled cellular structures, known for their high energy absorption capacity, are extensively utilized in transportation, aerospace, and other fields [19]. Common configurations include thin-walled columns [20,21], sandwich panels with cellular cores [22,23], graded polygons [24], and honeycomb structures [25,26,27]. AM technology facilitates the fabrication of these thin-walled cellular structures. Unlike metal or polymer AM, CFRTPCs AM requires continuous printing paths [28,29] to ensure part quality and dimensional accuracy. Consequently, path planning is a critical aspect of CFRTPC additive manufacturing, particularly for thin-walled cellular structures, which has stimulated extensive research [19,30,31]. The use of continuous fibers in CFRTPCs AM for thin-walled cellular structures often necessitates multiple filament cuts during printing [32]. Furthermore, even with continuous paths, excessive accumulation of thermoplastic filament can occur at path intersections, leading to uneven surfaces or printing failures [33]. Additionally, tension effects during AM can cause shape inaccuracies, twisting, folding, and misalignment of continuous fibers at sharp turns, adversely affecting printing accuracy and quality [32,34].

To address these issues, various solutions have been proposed. Zeng et al. [35] utilized fused deposition modeling with a nozzle for impregnating thermoplastic filament with reinforced fiber to fabricate honeycombs using pre-defined paths. Zhang et al. [36] introduced a jump point cutting algorithm to identify cutting points in CFRTPCs based on G-code jump commands. Huang et al. [37] proposed a multi-scale design and manufacturing strategy involving parallel optimization of micro-fiber orientation and macro-structural topology for CFRTPCs, generating printing paths by combining fiber orientation with Hamilton paths. However, these methods either rely on specific predefined paths or introduce fiber breakpoints to complete the printing process. Prashant et al. [38] developed a continuous path algorithm centered on constructing new Euler circuits to traverse all edges, requiring all vertices to have even degrees. This approach, however, introduces additional vertices and edges to form Euler circuits, potentially causing significant deviations from the target geometry. Wang et al. [39] proposed an optimized path planning method based on the Chinese postman problem from graph theory, treating each model slice as a graph structure and constructing Euler circuits by deleting and adding edges using Hierholzer’s algorithm. This method also necessitates adding redundant edges. Kohei et al. [40] generated Euler circuits and applied Hierholzer’s algorithm with non-crossing constraints for single-stroke AM of CFRTPCs, effectively avoiding path crossings but not addressing sharp turns. Huang et al. [41] optimized printing paths by searching for continuous paths on the dual graph, ensuring edges are traversed 1–2 times, and achieving a minimal total turning angle. While this method effectively addresses path continuity and turning angles, the generated paths contain unavoidable crossings that impair printing accuracy.

In light of the aforementioned discussions, this paper proposes a Euler graph-based path planning method for the additive manufacturing of CFRTPC thin-walled cellular structures. The primary objectives are to ensure path continuity to prevent fiber cutting, improve the surface quality of printed pieces, and validate the algorithm using a robot-assisted additive manufacturing system.

The proposed methodology involves several steps: First, the skeleton of the thin-walled cellular structure model is extracted and converted into a graph structure via data processing. Non-Eulerian graphs are then transformed into Euler graphs through edge doubling. Finally, an optimized Hierholzer’s algorithm generates printing paths that satisfy continuity, non-crossing, and sharp turn minimization criteria. The quality of the generated paths is evaluated based on the average turning angle and the proportion of non-sharp turns. To enhance printing accuracy at path intersections, a method for constructing pseudo-intersection nodes is introduced to maintain consistent layer height for each Euler circuit. The paths are subsequently validated using custom-built robot-assisted additive manufacturing system.

## 2. Materials and Methods

### 2.1. Algorithm Overview

**Figure 2:**
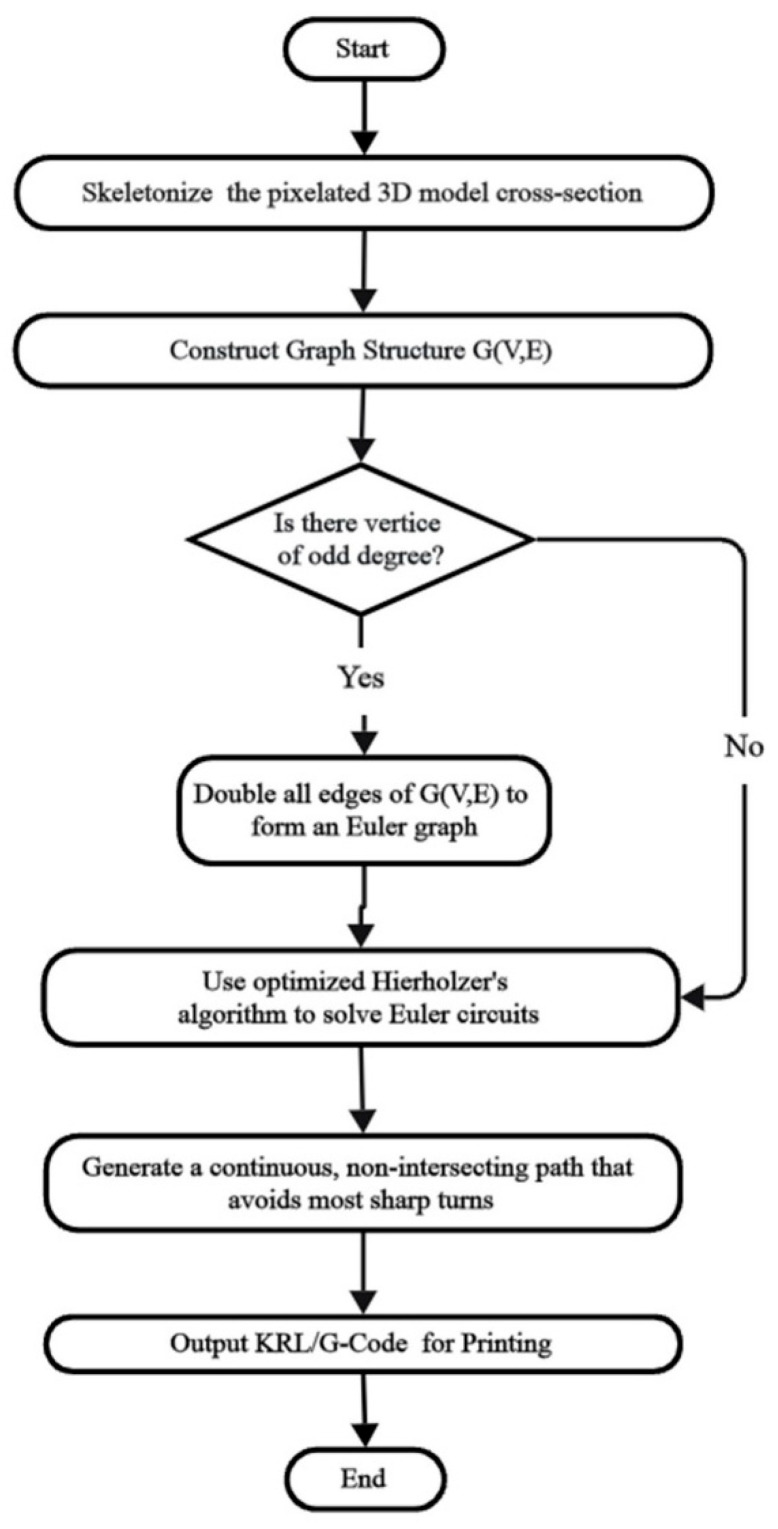
Flowchart of the proposed Euler graph-based path planning algorithm.

As depicted in Figure 2, the proposed algorithm proceeds as follows: First, the pixelated cross-section of the 3D model is skeletonized using the Zhang–Suen boundary fuzzy algorithm [42] to extract vertex and edge information, forming the graph structure G(V,E). Second, the degree of each vertex in $G\left(V,E\right)$ is assessed. If any vertex has an odd degree, all edges in G(V,E) are doubled to form a Euler graph. If all vertices have even degrees, the graph remains unaltered. Finally, Euler circuits are solved using an optimized Hierholzer’s algorithm. To prevent path crossings, a constraint is applied during algorithm traversal to search only among adjacent edges. To minimize sharp turns, the edge corresponding to the larger angle is selected from the candidate adjacent edges. The resultant paths satisfy continuity, non-crossing, and a low proportion of sharp turns. The output path files are subsequently converted into KUKA Robot Language (KRL) or G-code for actual printing.

### 2.2. Construction of Euler Graph

**Figure 3:**
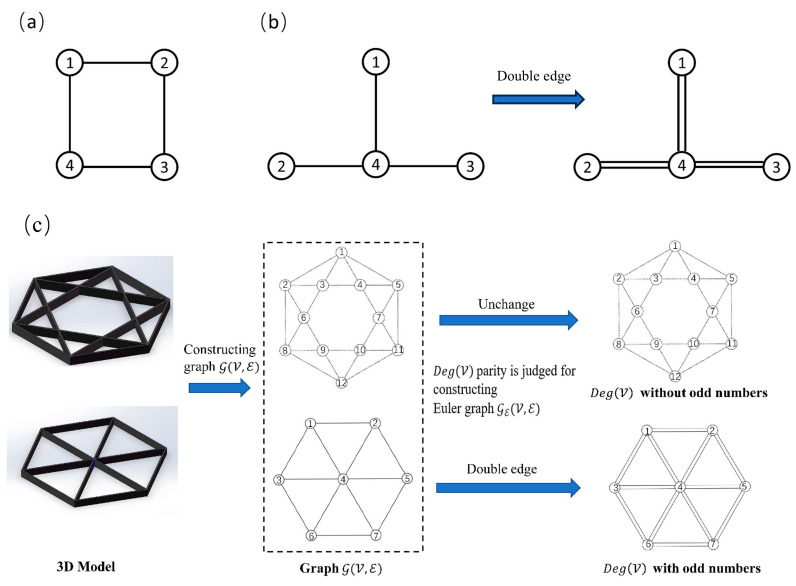
Construction of Euler graph: (**a**) Euler graph (rectangular loop); (**b**) non-Eulerian graph (T-shaped structure) converted to Euler graph via edge doubling; (**c**) construction of Euler graph from the solid model.

Achieving excellent mechanical properties in additively manufactured thin-walled cellular structures necessitates continuous printing paths. Conventional path generation methods often incorporate jump points, compromising the forming quality. This study proposes a novel method, illustrated in Figure 3c, which converts the solid model into a graph structure for path generation. The CAD model is first pixelated. The pixelated sections are then refined using a boundary fuzzy algorithm. Finally, an undirected graph G(V,E) is constructed based on the skeleton pixel vertices, where V and E represent the sets of vertices and edges, respectively.

If G(V,E) is the Euler graph, then there exists a Euler circuit. A Euler circuit refers to a path that traverses each edge of the graph exactly once, starts and ends at the same vertex. The existence of a Euler circuit is key to achieving a continuous and jump-free printing path. However, not all graph structures generated from thin-walled cellular structures have Euler graph characteristics. To construct a Euler circuit, the degree of each vertex deg(V) needs to be judged. The deg(V) is the number of adjacent edges of each vertex in the graph structure. A sufficient condition for the existence of Euler circuits in G(V,E) is that the degree of each vertex is even. When deg(V) of each vertex in the graph structure is even, the graph remains unchanged. In Figure 3a, the rectangular loop has four vertices (1, 2, 3, 4). The deg(V) of each vertex is 2 (an even number). Therefore, it is a Euler graph. A Euler circuit can be easily found, for example, 1 → 2 → 3 → 4 → 1. This path traverses all four edges exactly once without repetition, perfectly corresponding to a continuous printing path. However, even if there is a deg(V) with an odd number, some additional measures need to be taken to form a Euler graph. This problem can be solved by doubling all edges E in the graph structure. Then, the deg(V) of each vertex must be even and the modified graph is also a Euler graph $G_{E}(V,E)$. Figure 3b contains four vertices, each with an odd degree. Therefore, the graph is non-Eulerian. After applying the edge-doubling method, the original edges are doubled, resulting in six edges. Subsequently, the deg(V) of vertices 1, 2, and 3 become 1 × 2 = 2 (even), and the deg(V) of vertex 4 becomes 3 × 2 = 6 (even). Thus, the modified graph qualifies as a Euler graph, enabling the identification of a Euler circuit.

During the printing of CFRTPCs, fiber cutting significantly compromises the mechanical properties of the printed parts. By constructing a Euler graph through edge doubling, we ensure the existence of a continuous path (Euler circuit) that enables the entire structure to be printed in a single pass without jumps.

### 2.3. Solution of Euler Graphs

**Figure 4:**
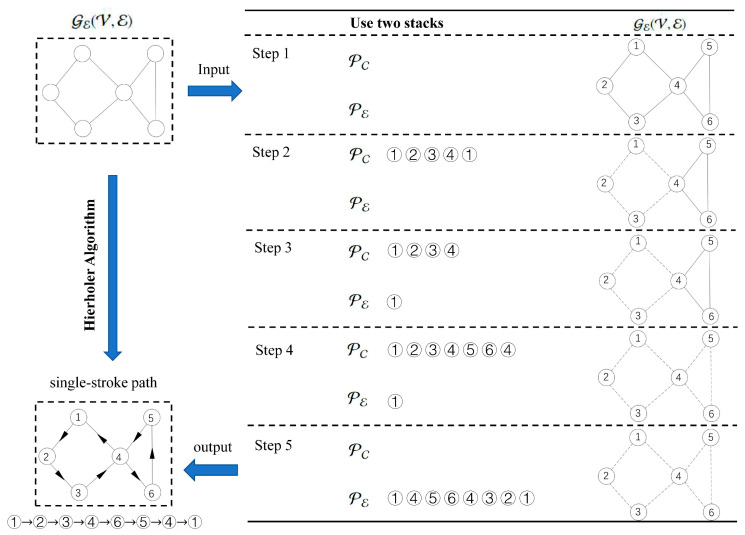
Hierholzer’s algorithm.

There are multiple methods for solving the Euler graph, such as Fleury’s algorithm, deep first search algorithm, and Hierholzer’s algorithm. Hierholzer’s algorithm is a very efficient method for finding Euler circuits. Firstly, two empty stacks $P_{C}$ and $P_{E}$ are initialized. In $G_{E}(V,E)$, a random vertex $v_{s}$ is chosen as the starting point of the algorithm. Next, a depth-first search is performed first from $v_{s}$. During searching process, each edge that has traversed is deleted and each vertex $v_{i}$ that has traversed is pushed into the stack $P_{C}$. If the current vertex $v_{i}$ has no adjacent edges, $v_{i}$ is removed from the $P_{C}$ and pushed to the $P_{E}$. If $P_{C}$ is empty, the algorithm ends. Otherwise, the algorithm will be backtracked and the depth-first search continues. Finally, the vertices in $P_{E}$ are popped in the reversed order. The sequence is the Euler circuit in the $G_{E}(V,E)$. The specific process is shown in Figure 4.

More details are shown in Algorithm 1. An optimized Hierholzer’s algorithm is used in this paper. The time complexity of Hierholzer’s algorithm is O(n). It has significant advantages in solving the Euler circuit for a complex graph.
**Algorithm 1:** Hierholzer’s Algorithm**Input**: Undirected graph $G_{E}(V,E)$ **Output:** Euler circuit $P_{E}$    1: **function** Circular Path( Adjacent vertex list ${List}_{a}$, Start vertex $v_{s}$)    2:    Find an adjacent vertex $v_{n}$ of $v_{s}$ and remove $v_{s}$ from ${List}_{a}$[$v_{n}$]    3:    Creat a stack $P_{C}\;\leftarrow v_{s},v_{n}$    4:    **while** $v_{s}$ and $v_{n}$ are unequal **do**    5:     Creat a stack $v_{c}\;=\;v_{n}$    6:     Find an adjacent vertex $v_{n}$ of $v_{c}$ and remove $v_{c}$ from ${List}_{a}$[$v_{n}$]    7:     $\;P_{C}\;\leftarrow v_{n}$    8:    **end while**    9:    **return**
$P_{C}$    10: **end function**    11:    12: **function** EulerCircuit($G_{E}(V,E)$)    13:     Creat Adjacent vertex list ${List}_{a}$ from $G_{E}(V,E)$    14:     $P_{E}\;=$ CircularPath(${List}_{a}\;$, $v_{s}$) // $v_{s}$ is a random vertex in V    15:     **for** Check each vertex in V **do**    16:      **if** Have any ${List}_{a}$[$v_{i}$] ≠∅ **then**    17:      ${P^{\prime }}_{E}\;=$ Circular Path(${List}_{a}\;$, $v_{i}$)    18:       Insert ${P^{\prime }}_{E}$ in reverse order at ${P^{\prime }}_{E}$ of $v_{i}$    19:      **end if**    20:     **end for**    21:     **return**
$P_{C}$    22: **end function**

**Figure 5:**
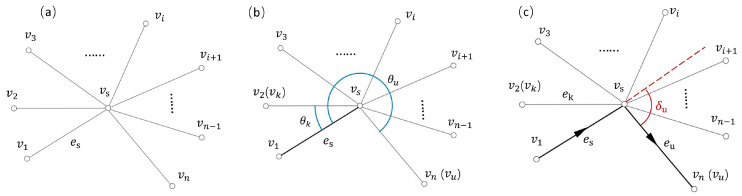
Constraints on Hierholzer’s algorithm: (**a**) the vertices neighboring to $v_{s}$; (**b**) the minimum angle $\theta_{k}$ and the maximum angle $\theta_{u}$; (**c**) finding the minimum turning angle $\psi_{u}$.

Although the Hierholzer’s algorithm could efficiently generate Euler circuits, it cannot guarantee the minimum of the path crossing and sharp turn problems. In this paper, an optimized Hierholzer’s algorithm with constraints was proposed to improve printing quality. In $G_{E}(V,E)$, we could give priority to avoiding crossing in the path and minimizing the number of sharp turns. First, vertex $v_{s}$ and a previous edge $e_{s}$ are known, as shown in Figure 5a. Second, $e_{i}$ is the edge connected by $v_{s}$ and $v_{i}$. Finally, $\theta_{i}$ is the angle formed by $e_{i}$ and $e_{s}$. The computation of $\theta_{i}$ follows in a clockwise or counterclockwise direction, as shown in Equation (1). All the vertices neighboring to $v_{s}$ are traversed, and Ω is the set of all $\theta_{i}$. The definition of Ω is shown in Equation (2). It is not difficult to introduce the correspondence between $\theta_{i}$ and $v_{i}$ by Equation (1). The vertices $v_{k}$ and $v_{u}$ correspond to the minimum angle $\theta_{k}$ and the maximum angle $\theta_{u}$, as shown in Equation (3) and Figure 5b. $\psi_{i}$ is the turning angle formed by $\vec{e_{s}}$ and $\vec{e_{i}}$, as shown in Equation (4). Afterwards, the vertex $v_{n}$ corresponding to the larger of the angles $\psi_{k}$ and $\psi_{u}$ is searched, as shown in Equation (5) and Figure 5c. More details are shown in Algorithm 2 non-crossing path algorithms for optimizing turning-Angle. The accuracy of the printed part is improved by avoiding crossings in the path and minimizing the number of sharp turns during the solution of the Euler circuits by this approach.

(1) $$\theta_{i}=\{\begin{array}{c} \arccos\frac{\overset{\to }{{-e}_{s}}\cdot \overset{\to }{e_{i}}}{\left|-\overset{\to }{e_{s}}\right|\cdot \left|\overset{\to }{e_{i}}\right|}\left(-\overset{\to }{e_{s}}\times \overset{\to }{e_{i}}\geq 0\right)\\ 2\pi -arccos\frac{\overset{\to }{{-e}_{s}}\cdot \overset{\to }{e_{i}}}{\left|\overset{\to }{{-e}_{s}}\right|\cdot \left|\overset{\to }{e_{i}}\right|}(-\overset{\to }{e_{s}}\times \overset{\to }{e_{i}}<0) \end{array},\theta_{i}\in \left[0,2\pi \right]$$

(2) $$\Omega ={\left\{\theta_{i}\right\}}_{i=1}^{n}=\{\theta_{1},\theta_{2}\cdots ,\theta_{n}\}$$

(3) $$\theta_{k}=min\;\Omega ,\theta_{u}=max\;\Omega$$

(4) $$\psi_{i}=arccos\frac{\overset{\to }{e_{s}}\cdot \overset{\to }{e_{i}}}{\left|\overset{\to }{e_{s}}|\cdot |\overset{\to }{e_{i}}\right|}$$

(5) $$v_{n}=\{\begin{array}{c} v_{k}\;(\psi_{u}>\psi_{k})\\ v_{u}\;(\psi_{u}\leq \psi_{k}) \end{array}$$

**Algorithm 2:** Non-crossing path algorithms for optimizing turning-Angle    1: **function** OptimizationPath(Adjacent vertex list ${List}_{a}$, Start vertex $v_{s}$, Start edge $e_{s}$)    2:   **for** Check each vertex $v_{i}$ in ${List}_{a}$[$v_{s}$] **do**    3:     $\;e_{i}$ is the edge formed by $v_{s}$ and $v_{i}$    4:     **if**
$\vec{e_{s}}\;\times \;\vec{e_{i}}\;\geq 0$
**then**
$\theta_{i}\;=$ $\arccos\frac{\overset{\to }{{-e}_{s}}\cdot \overset{\to }{e_{i}}}{\left|-\overset{\to }{e_{s}}\right|\cdot \left|\overset{\to }{e_{i}}\right|}\;$    5:     **else**
$\theta_{i}\;=2\pi \;-$ $\arccos\frac{\overset{\to }{{-e}_{s}}\cdot \overset{\to }{e_{i}}}{\left|-\overset{\to }{e_{s}}\right|\cdot \left|\overset{\to }{e_{i}}\right|}$    6:     **end if**    7:      $\;\Omega \;\leftarrow \theta_{i}$    8:   **end for**    9:   $\theta_{k}=min\;\Omega ,\theta_{u}=max\;\Omega$    10:  $v_{k}\;=\;f\left(\theta_{k}\right)\;,v_{u}\;=\;f\left(\theta_{u}\right)$    11:   $\psi_{i}=arccos\frac{\overset{\to }{e_{s}}\cdot \overset{\to }{e_{i}}}{\left|\overset{\to }{e_{s}}|\cdot |\overset{\to }{e_{i}}\right|}$    12:   **if**
$\psi_{k}\;\geq \;\psi_{u}$
**then**
$v_{n}\;=\;v_{u}$    13:   **else**
$v_{n}\;=\;v_{k}$    14:   **end if**    15:   Creat a stack $P_{C}\;\leftarrow v_{s},v_{n}$    16:   **while**
$v_{s}$ and $v_{n}$ are unequal **do**    17:   Creat a vertex $v_{c}\;=\;v_{n}$    18:     Find an adjacent vertex $v_{n}$ of $v_{c}$ and remove $v_{c}$ from ${List}_{a}$[$v_{n}$]    19:   $P_{C}\;\leftarrow v_{n}$    20:   **end while**    21:   **return**
$P_{C}$    22: **end function**

## 3. Results and Discussion

### 3.1. Robot-Assisted Additive Manufacturing System

**Figure 6:**
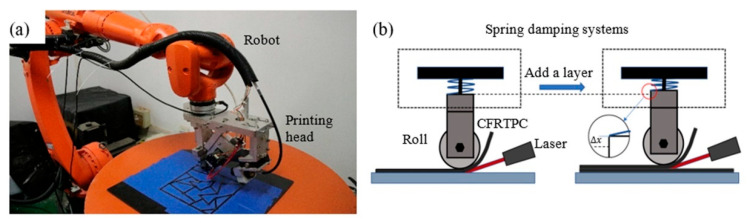
Robot-assisted additive manufacturing system: (**a**) robot-assisted additive manufacturing equipment; (**b**) height variation in the pressure roller during double-layer printing within a single loop.

The method mentioned was verified by a custom-built robot-assisted additive manufacturing system consisting of KUKA KR20 industrial robot (KUKA KR20 industrial robot made from the KUKA corporation, Augsburg, Germany), as shown in Figure 6. A roller spring damping unit was installed on the printing head of this equipment. The roller could be used to generate pressure to flatten continuous fiber composite filaments which were heated by the laser beam. High-density parts were formed through melting and layer-by-layer stacking. In this algorithm, a Euler circuit path may consist of double layers. When the second layer was printing, the spring damping system was compressed. The amount of spring compression was related to the layer height. Δx was a single layer height, and the thickness was about 0.06 mm applying 16 N pressure by roller compaction. Before printing, a preheating device at the wire feeding nozzle was used to raise the temperature of the prepreg wire. The prepreg wire was made of continuous carbon fiber covered by poly lactic acid (PLA). The robot end positions were calculated according to the printing path information generated by our algorithm. Other process parameters were also entered into the robot-assisted additive manufacturing system.

### 3.2. Visualization of Generated Paths

**Figure 7:**
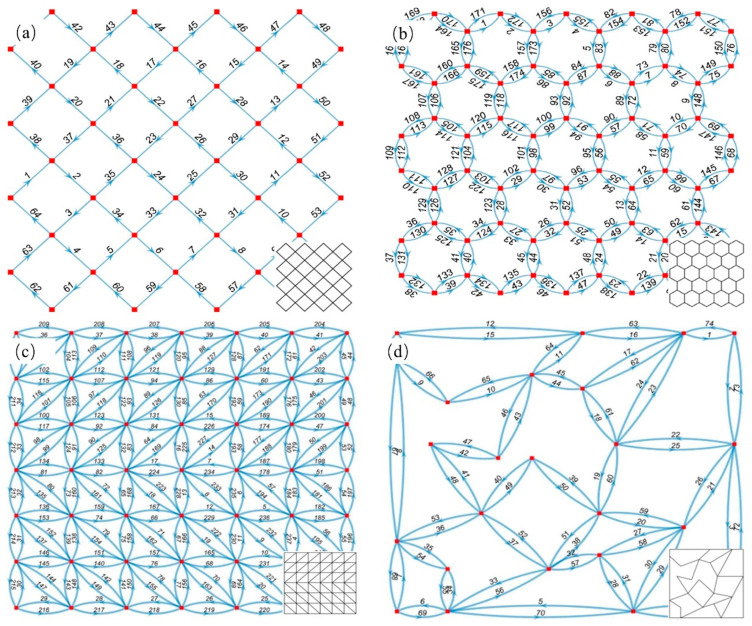
Visualization of generated paths: (**a**) Quadrilateral grid structure; (**b**) Honeycomb structure; (**c**) Truss structure; (**d**) RTWCS.

As shown in Figure 7, various different thin-walled cellular structures were tested to verify the feasibility and robustness of the algorithm. The quadrilateral grid structure, honeycomb structure, truss structure, and random thin-walled cellular structure (RTWCS) were selected. For instance, Figure 7b displays the undirected graph derived from the CAD model and the resulting directed graph. Red points indicate vertices V. Curved directed segments represent the path direction for clarity regarding the double-layer single-loop path; the actual path is the straight line between vertices. Traversal order is indicated by serial numbers adjacent to segments. The quadrilateral grid structure in Figure 7a, after conversion to a graph structure, was identified as a Euler graph, enabling direct generation of a single-layer, single-loop path. Conversely, the honeycomb, truss, and RTWCS in Figure 7b–d do not satisfy Euler circuit conditions, necessitating the generation of double-layer, single-loop paths.

### 3.3. Quality Evaluation

**Figure 8:**
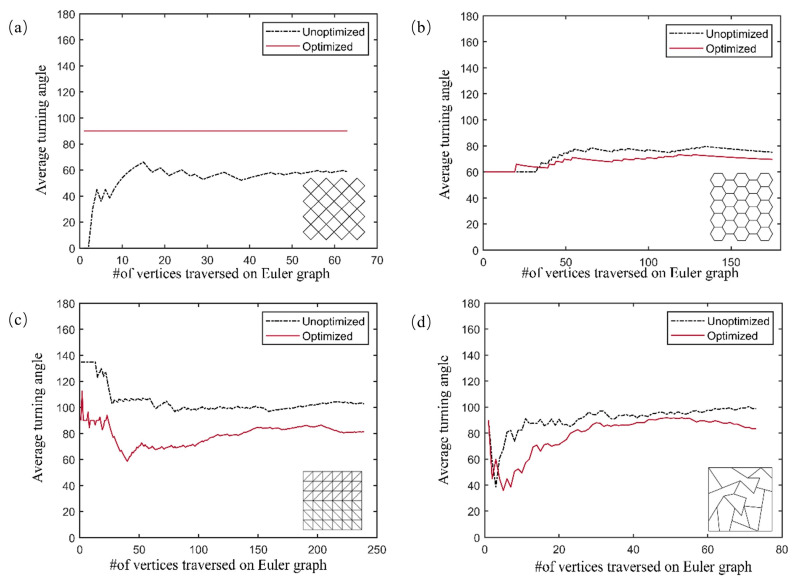
Variation in average turn angle with the number of traversed vertices: (**a**) Quadrilateral grid; (**b**) Honeycomb; (**c**) Truss; (**d**) RTWCS.

Sharp turns may lead to shape inaccuracies, twisting, folding, and misalignment in CFRTPCs additive manufacturing. An average turning angle of 120° is considered a critical threshold [34]. Therefore, in this work, the average turning angle and the percentage of turning angle less than 120° were used to evaluate the quality of the algorithm. The results are shown in Table 1 and Figure 8.

Although the average turning angle is lower in the unoptimized path for quadrilateral grids, there is a large number of intersecting lines. In contrast, all paths generated by the optimized Hierholzer’s algorithm avoid crossings and maintain continuity, which is beneficial for CFRTPCs AM. Compared to unoptimized paths, the optimized algorithm reduced the average turning angle for the honeycomb structure from 75.81° to 66.85° (an 11.82% reduction), thereby decreasing the number of U-turns and improving printing accuracy. The optimized algorithm was also validated on the truss structure and RTWCS, reducing average turning angles by 20.88% and 15.54%, respectively. The variation in average turn angle with the number of traversed vertices is shown in Figure 8. The optimized paths exhibited no crossings or overlaps compared to the unoptimized Hierholzer’s algorithm. Furthermore, the percentages of turns less than 120° for most models were significantly improved by the optimized algorithm, aiding in the reduction in fiber twisting, misalignment, and breakage caused by sharp turns.

### 3.4. Experiment Analysis

**Figure 9:**
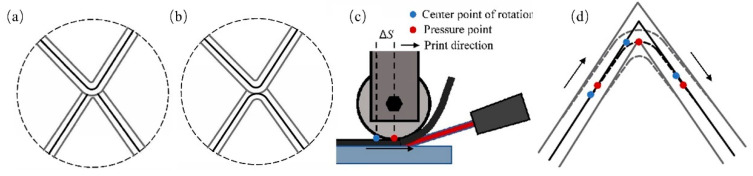
Pseudo-intersection node generation: (**a**) overlapping intersection node; (**b**) pseudo-intersection node; (**c**) schematic diagram of ΔS; (**d**) path morphology while ΔS≠0.

When traversing Euler circuits, each edge is traversed the same number of times. However, if a vertex is shared with multiple edges, the number of traversals at some vertices is not always the same as the number of traversals of the edges, leading to thermoplastic filament accumulation at the vertex. This results in greater thickness at vertices compared to edges, compromising manufacturing precision or causing processing failures. To solve this problem, the path is slightly offset at corners by adjusting the process parameters. As shown in Figure 9a,b, filament overlap can be effectively reduced when a vertex is traversed twice during printing. Through experimental investigation, a pseudo-intersection node was proposed to realize a reasonable deviation at corners. As shown in Figure 9c, the yaw axis distance ΔS between the rotating center and pressure point at the printing head can be adjusted while a turn is taking place. The center point of rotation coincides with pressure point if ΔS=0. The actual printing path will coincide exactly with the designed printing path. As shown in Figure 9d, the trajectory will be deviated when ΔS≠0. The value of ΔS can be adjusted according to different angular values for a turn. It can avoid thermoplastic filament aggregation at intersections. Thickness in the vertex remains consistent with that in the edges. Printing quality can also be effectively improved.

To verify the reliability of constructing pseudo-intersection nodes, experiments tested printing fiber intersection nodes with various angular turning angles. The typical used model includes forming 60/90/120° turning angles of the filaments and their intersecting nodes, with 1.5/2/3 times as many traverses of the nodes per printed layer as the number of traverses of the single-wall formed segments. All models were printed with consistent process parameters. A wall with a total thickness of 6 mm was fabricated by the robot using layer thickness of 0.08 mm over the course of 75 layers. Roughness Ra and depth images were measured using the super depth-of-field digital microscope system (VHX-1000C made from the KEYENCE corporation, Osaka, Japan). The KEYENCE VHX-1000C is a high-performance digital microscopy system that surpasses the limitations of conventional optical microscopy. For each typical specimen, printing tests were conducted under five different ΔS conditions, and the Ra values were calculated based on three repeated measurements. w was measured in proportion to the thickness of the single-walled fibers using vernier calipers.

**Figure 10:**
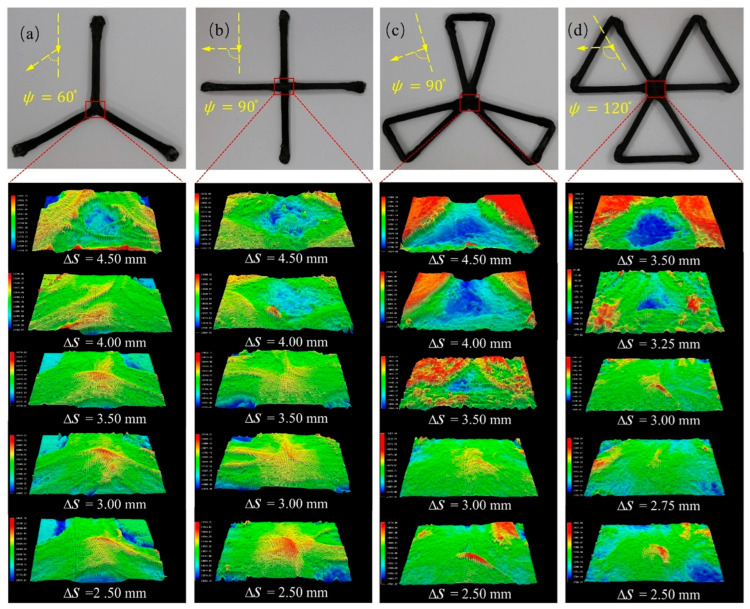
Ultra-depth-of-field test results at typical specimen nodes for different yaw axis offset distances (corresponding to the data in Table 2): (**a**) 60° turning angle and k=1.5, (**b**) 90° turning angle and k=2, (**c**) 90° turning angle and k=3, (**d**) 120° turning angle and k=3.

Figure 10 illustrates the impact of different yaw axis distances ΔS on the forming accuracy at the nodes under the same forming temperature and pressure. Generally, small ΔS values result in excessive overlap at the nodes, causing protrusions. As ΔS increases, the node filling becomes sparser, and node thickness decreases due to the dispersion of the thermoplastic filament. This is attributed to the growing disparity between the actual and target paths. Variations in thickness and defects caused by fiber path crossings and overlaps are a recognized challenge in additive manufacturing. For instance, in continuous fiber 3D printing, Yu et al. [33] reported increased thickness and surface irregularities due to fiber stacking at path intersections. This study aims to mitigate such defects by adjusting ΔS. Further details on the average roughness Ra and w variations with yaw axis offset distance ΔS are provided in Table 2.

For the node with a 60° turning angle shown in Figure 10a, the model achieves the flattest and least rough nodes at ΔS=4.0 mm, with an average Ra of 62 µm and w of 99.68%. When ΔS is less than 2.5 mm, all specimens fail to print in under 60 layers due to the excessive towpreg offset under the roller at the forming corner. This causes the filament to be out of the laser radiation area or the pressure roller range. On the other hand, when ΔS exceeds 3.5 mm, the test model in Figure 10d fails to form bonded nodes due to sparse paths. When ΔS surpasses 4.5 mm, even the test models in Figure 10a–c fail to form nodes.

The ratio of the number of node traversals to the number of linear segment traversals in each layer is denoted as k. Aside from the yaw axis offset distance ΔS, the value of k also influences forming quality. The models in Figure 10b,c both feature a 90° turn angle, with k values of 2 and 3, respectively. Comparing the test data for these two models in Table 2, the effect of ΔS on w is relatively small for the model with k=2. As ΔS decreases from 3.5 mm to 2.5 mm, w only increases from 97.03% to 107.2%, a difference of approximately 10%. In contrast, for the model with k=3, a slight change of around ±0.5 mm around 3 mm causes different forming outcomes, resulting in a w change of 81%. It can be deduced that when the corner angle is constant, larger k values lead to greater sensitivity of w to ΔS changes. Therefore, when designing a path-filling strategy with nodes, it is important to avoid multiple objective paths intersecting at a single node.

With a fixed ΔS, different corner angles ψ of the target path yield varying effects on the actual path bias, with larger ψ values leading to greater actual path bias. For instance, comparing the models in Figure 10c,d, with ΔS > 3.5 mm, the model with a 120° turn angle fails to form the node due to no intersection, while the model with a 90° turn angle can still form the node. When ΔS = 3 mm, the w for the 120° turn angle model is 100.2%, while for the 90° turn angle model, it is 105.6%. This is consistent with the mechanism of forming corners using fused filament fabrication (FFF). As the towpreg flattens, the towpreg is already formed and held by the adhesive forces of the printing platform or the part’s surface, which causes a pultrusion force on the unformed towpreg, resulting in a pultrusion effect, leading to twisting and misalignment of the towpreg, which is more pronounced with larger forming corners. As ψ increases, the actual offset of fibers at the corners increases, leading to greater difficulty in node formation. This phenomenon is consistent with the conclusion proposed by Zhang et al. [34] in their study on fiber misalignment and breakage.

Under the same k and ΔS conditions, the Ra of the 120° turn angle is slightly better than that of the 90° turn angle. A larger turn angle provides a gentler turning path, offering more geometric flexibility for material redistribution. 

As shown in Table 2, the lowest Ra values consistently occur when w is close to 100%. This strongly demonstrates that thickness uniformity is a prerequisite for controlling surface roughness. When w is significantly below 100%, it indicates insufficient material at the intersections, resulting in depressions that increase Ra. Conversely, when w is significantly above 100%, it suggests material accumulation forming protrusions, which also elevates Ra. Therefore, by adjusting ΔS to bring w close to 100%, the primary surface defects caused by uneven material volume distribution are essentially eliminated. This reduction in defects allows Ra to approach its theoretical minimum.

Adjusting the yaw axis distance during cornering helps correct the real path offset caused by the pultrusion force. An optimal yaw axis distance for nodes with varying angular corners minimizes roughness while maintaining a flat structure. Process know-how regarding node forming precision can guide the creation of additive manufacturing pathways. The best yaw axis distance for each corner can be stored in a database and integrated into robotic system. This way, it can be dynamically utilized during printing to optimize node accuracy for the generated part.

**Figure 11:**
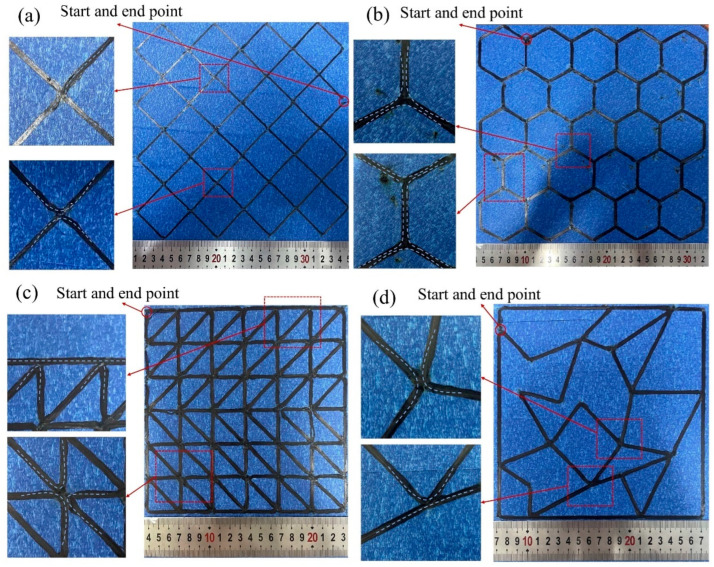
Actual printing of thin-walled cellular structures: (**a**) Quadrilateral grid; (**b**) Honeycomb; (**c**) Truss; (**d**) RTWCS.

Actual printing of quadrilateral grid, honeycomb, truss, and RTWCS were performed using robot-assisted additive manufacturing equipment to verify the method mentioned. The roller pressure was set to 16N  and the laser power was set to 11.5 W As shown in Figure 11, the printing paths generated by the optimized Hierholzer’s algorithm exhibit characteristics of no crossings, full coverage, and continuity. Furthermore, the paths contain few sharp turns. The geometric inaccuracies and fiber filament defects of the printed parts mainly occurred in the unavoidable sharp-turns of the paths. In addition, the method used in this paper relies on the double layers with a single loop in the path. The problem that the number of traversals of vertices is different from the edges is unavoidable. In the actual printing, the relationship between ΔS and ψ was obtained experimentally. A pseudo-intersection was constructed to avoid the bumps of the printed parts due to the overlapping of filaments. The reasonable pseudo-intersection ensures the realization of the layer-by-layer printing process. Herein, the effect of printing parameters for constructing pseudo-intersections was just simply explored, as shown in Figure 11. It was found that by controlling ΔS could construct pseudo-intersections without bumps and gaps. Therefore, the key to acquiring the highly accurate printed parts is to design the printing parameters dynamically for different turning angles.

**Figure 12:**
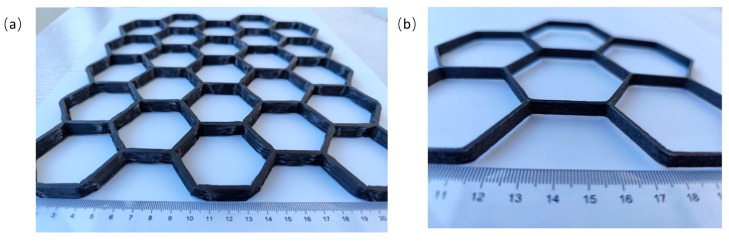
Multi-layer actual printing of honeycomb structures of CFRTPCs: (**a**) made by the robotic-assisted additive manufacturing system; (**b**) made by the FDM system.

Robotic-assisted additive manufacturing system for CFRTPCs and FDM system were used to conduct multi-layer actual printing of honeycomb structures, respectively, to validate the method described in this paper. The model in Figure 12a was fabricated by converting the path into KRL code for the robot-assisted additive manufacturing system, with a layer thickness of 0.08 mm and an overall model thickness of 10 mm. In addition, the model in Figure 12b was produced by converting the path into G-code for the FDM system, with a layer thickness of 0.15 mm and an overall thickness of 6 mm. Both fabricated honeycomb structures exhibit the characteristics of being non-crossing and continuous, thereby validating the robustness of the algorithm and ensuring its reliability and practicality.

## 4. Conclusions

This paper proposed a Euler graph-based path planning method for the additive manufacturing of continuous fiber-reinforced thin-walled cellular structures. Euler graphs were constructed by doubling each edge and optimizing turning angles. An optimized Hierholzer’s algorithm was employed to generate printing paths by constructing Euler circuits. The method effectively avoids filament crossings and reduces the number of sharp turns, thereby mitigating fiber twisting, misalignment, and breakage. The optimized Hierholzer’s algorithm reduced the number of average turns and the percentage of turning angles to less than 120° while avoiding crossing in the path. The average turning angle was reduced by up to 20.88% and the number of turning angles less than or equal to 120° increased by up to 26.67% using an optimized algorithm. A method of constructing pseudo-intersections was proposed to solve the bumps caused by thermoplastic filament enrichment in vertices. Paths for some thin-walled cellular structures of CFRTPCs were successfully validated using a robot-assisted additive manufacturing system.

The proposed path planning method provides a basis for the additive manufacturing of thin-walled cellular structures of CFRTPCs. However, it is important to note that the method does not guarantee a globally optimal solution with the minimum total turning angle for the printing path. Future work will focus on further optimizing the algorithm to better satisfy the requirement for minimizing the total turning angle.

## Figures and Tables

**Table 1:** Statistical comparison of unoptimized paths and optimized paths.

Models	Average Turning-Angle (Deg)		Percentage of Turning Angles ≤ 120°	
	Unoptimized	Optimized	Unoptimized	Optimized
Quadrilateral grid	58.57	90	0	0
Honeycomb	75.81	66.85 (↓ 11.82%)	87.43	92 (↑ 5.227%)
Truss	102.8	81.34 (↓ 20.88%)	53.56	64.85 (↑ 21.08%)
RTWCS	98.84	83.48 (↓ 15.54%)	61.64	78.08 (↑ 26.67%)

**Table 2:** Ultra-depth-of-field test results in typical specimen nodes for different yaw axis offset distances.

Node Test Model	ψ (°)	k	ΔS (mm)	Average Calculated Ra (μm)	w
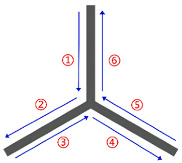	60	1.5	4.50	0.17 ± 0.03	66.56%
			4.00	0.06 ± 0.02	99.68%
			3.50	0.11 ± 0.05	103.2%
			3.00	0.13 ± 0.03	108.6%
			2.50	0.11 ± 0.03	113.9%
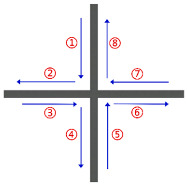	90	2	4.50	0.34 ± 0.17	62.70%
			4.00	0.21 ± 0.08	78.39%
			3.50	0.10 ± 0.04	97.03%
			3.00	0.11 ± 0.06	107.2%
			2.50	0.12 ± 0.06	126.1%
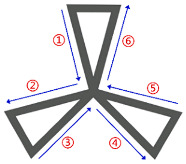	90	3	4.50	0.2 ± 0.06	27.23%
			4.00	0.22 ± 0.09	39.36%
			3.50	0.24 ± 0.04	49.33%
			3.00	0.09 ± 0.02	105.6%
			2.50	0.10 ± 0.02	130.6%
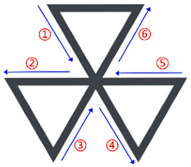	120	3	3.50	0.22 ± 0.05	35.80%
			3.25	0.19 ± 0.06	59.36%
			3.00	0.08 ± 0.02	100.2%
			2.75	0.08 ± 0.02	121.9%
			2.50	0.10 ± 0.03	128.5%

## Data Availability

The original contributions presented in this study are included in the article. Further inquiries can be directed to the corresponding authors.
